# Shifts in sensitivity of amphibian metamorphosis to endocrine disruption: the common frog (*Rana temporaria*) as a case study

**DOI:** 10.1093/conphys/coaa100

**Published:** 2020-12-14

**Authors:** Katharina Ruthsatz, Kathrin H Dausmann, Katharina Paesler, Patricia Babos, Nikita M Sabatino, Myron A Peck, Julian Glos

**Affiliations:** 1 Institute of Zoology, Universität Hamburg, Martin-Luther-King-Platz 3, 20146 Hamburg, Germany; 2 Zoological Institute, Technische Universität Braunschweig, Mendelssohnstraße 4, 38106 Braunschweig, Germany; 3 Department of Life Sciences, Hamburg University of Applied Sciences, Ulmenliet 20, 21033 Hamburg, Germany; 4 Institute of Marine Ecosystems and Fisheries Science, Universität Hamburg, Große Elbstraße 133, 22767 Hamburg, Germany; 5 Department of Coastal Systems, Royal Netherlands Institute for Sea Research, PO Box 59 1790, AB Den Burg, Netherlands

**Keywords:** *Amphibian conservation*, *energy budgets*, *environmental stress*, *global change*, *standard metabolic rate*, *stress physiology*

## Abstract

Effective conservation actions require knowledge on the sensitivity of species to pollution and other anthropogenic stressors. Many of these stressors are endocrine disruptors (EDs) that can impair the hypothalamus–pituitary–thyroid axis and thus alter thyroid hormone (TH) levels with physiological consequences to wildlife. Due to their specific habitat requirements, amphibians are often sentinels of environmental degradation. We investigated how altered TH levels affected the bioenergetics of growth and development (i.e. age, size, metabolism, cardiac function and energy stores) before, during and after metamorphosis in the European common frog (*Rana temporaria).* We also determined how ontogenetic stage affected susceptibility to endocrine disruption and estimated juvenile performance. TH levels significantly affected growth and energetics at all developmental stages. Tadpoles and froglets exposed to high TH levels were significantly younger, smaller and lighter at all stages compared to those in control and low TH groups, indicating increased developmental and reduced growth rates. Across all ontogenetic stages tested, physiological consequences were rapidly observed after exposure to EDs. High TH increased heart rate by an average of 86% and reduced energy stores (fat content) by 33% compared to controls. Effects of exposure were smallest after the completion of metamorphosis. Our results demonstrate that both morphological and physiological traits of the European common frog are strongly impacted by endocrine disruption and that ontogenetic stage modulates the sensitivity of this species to endocrine disruption. Since endocrine disruption during metamorphosis can impair the physiological stress response in later life stages, long-term studies examining carry-over effects will be an important contribution to the conservation physiology of amphibians.

## Teaser

We investigated how altered thyroid hormone levels affected the bioenergetics of growth and development in *Rana temporaria*. Our results demonstrate that both morphological and physiological traits of the European common frog are strongly impacted by endocrine disruption and that ontogenetic stage modulates the sensitivity of *R. temporaria* to endocrine disruption.

## Introduction

Global change is, in part, responsible for the alarming rates of local extinction through exposing wildlife to increased environmental variation and an array of chemical, physical and biological stressors (Noyes *et al.,* 2009; Dantzer *et al.,* 2014). Among all vertebrates, amphibians are particularly endangered (Collins, 2010; Hoffmann *et al.,* 2010). The loss of amphibian diversity around the world is due to multiple stressors associated with global environmental change (Stuart *et al.,* 2004; Strong *et al.,* 2017) such as habitat fragmentation, invasive species, chemical pollution, climate change and infectious diseases (Pacifici *et al*., 2015; Rollins-Smith, 2017; Scheele *et al.,* 2019). Furthermore, numerous environmental stressors have the ability to alter endocrine function (Carr and Patiño, 2011) and are thus characterized as endocrine disruptors (EDs) (Kloas and Lutz, 2006; Kloas *et al.,* 2009). Amphibian larvae are especially sensitive to EDs due to their highly permeable skin (Yu *et al.,* 2013; Strong *et al.,* 2017), their limited capacity for habitat selection (Sanzo and Hecnar, 2006; Yu *et al.,* 2013) and their critical hormone-regulated metamorphosis during early life (Searcy *et al.,* 2015).

Amphibian metamorphosis is mainly regulated by thyroid hormones (THs) (Furlow and Neff, 2006; Tata, 2006), which determine the developmental rate (Shi, 2000; Brown and Cai, 2007). Many EDs target the hypothalamus–pituitary–thyroid axis, which is required for production of THs (Carr and Patiño, 2011). A large number of aquatic contaminants such as pesticides and herbicides, road salt, fertilizers, heavy metals and active pharmaceutical ingredients have been shown to disrupt and inhibit the normal action of THs in amphibians, leading to changes in growth, development and metabolism (rev. in Mann *et al.,* 2009; Kashiwagi *et al.,* 2009; Carr and Patiño, 2011). Inhibition of TH production pathways results in decreased developmental rates (Carr *et al.,* 2003; Bulaeva *et al.,* 2015) with tadpoles metamorphosing at a larger size and older age (Shi, 2000).

Under natural conditions, stress associated with, e.g. exposure to road de-icing salt (Sanzo and Hecnar, 2006), desiccation risk (Gervasi and Foufopoulos, 2008) and heatwaves (Smith-Gill and Berven, 1979; reviewed in Ruthsatz *et al.,* 2018a) will accelerate metamorphosis as an adaptive stress response (Denver, 1997b; Mann *et al.,* 2009). This response is mediated through a cascade of signalling events activating the neuroendocrine stress axis (Mann *et al.,* 2009; Dantzer *et al.,* 2014). In most cases, activation of the neuroendocrine stress axis increases stress hormone levels (Denver, 1997a). These stress hormones also target the hypothalamus–pituitary–thyroid axis (Carr and Patiño, 2011) and may synergize with THs resulting in increased TH production (Glennemeier and Denver, 2002a, 2002b; Laudet, 2011; Kulkarni and Buchholz, 2012). Consequently, increase of TH production by environmental stressors is also a form of endocrine disruption (Mann *et al.,* 2009). Anuran larvae with high TH levels display increased developmental and metabolic rates and decreased growth rates (Rowe *et al.,* 1998; Brown and Cai, 2007), which results in shorter larval periods, smaller size at the onset of metamorphosis and higher energetic maintenance and developmental costs (Denver, 1998, 2009; Orlofske and Hopkins, 2009). Exposing tadpoles to exogenous THs is an established method to simulate the proximate effects of environmental stressors on the TH system (Denver *et al.,* 2002; Tata, 2006; Denver, 2009).

In all vertebrates, THs are not only critical for regulating growth and development, but also for regulating energy metabolism (Frieden, 1981; Sheridan, 1994; Choi *et al.,* 2017). If the TH concentration changes due to endocrine disruption by environmental stressors, a whole suite of physiological processes may be influenced (Hulbert and Else, 2004; Steyermark *et al.,* 2005; Ruthsatz *et al.,* 2018b, 2018c, 2019). Increased levels of THs increase the standard metabolic rate (SMR; measured as the rate of O_2_ consumption at rest) and resting heart rate (f_H_; Dillmann, 2002; Little and Seebacher, 2014a, 2014b). These changes result from increases in the activity of enzymes (Chiu and Woo, 1988; Rowe *et al.,* 1998) and increased density of mitochondria (Steyermark *et al.,* 2005) in metabolically relevant tissues (e.g. liver and red skeletal muscle). The SMR provides an estimate of the energy required to cover basic physiological functions (Rowe *et al.,* 1998; Beck and Congdon, 2003). In animals with a high SMR, less energy can be used or saved for physical performance or development (Steyermark *et al.,* 2005; Orlofske and Hopkins, 2009) and, also, oxidative stress might be increased (Burraco and Gomez-Mestre, 2016; Burraco *et al.,* 2017; Burraco *et al.,* 2020). As metamorphosis is an energy-consuming process (Sheridan and Kao, 1998; Beck and Congdon, 2003), a low SMR is expected to be advantageous to tadpoles. Tadpoles with larger energy storages at the onset of metamorphosis are more likely to successfully complete metamorphosis and become juvenile froglets with better body condition and higher survival rates (Orlofske and Hopkins, 2009). Therefore, the SMR before, during and after completion of metamorphosis has consequences not only for energy partitioning between growth, development and metabolism but also for life time fitness (Steyermark *et al.,* 2005; Muir *et al.,* 2014).

Amphibian metamorphosis is separated into premetamorphosis, prometamorphosis and metamorphic climax (Etkin, 1932; Gosner, 1960; Dodd and Dodd, 1976). These stages are associated with key developmental changes (Gosner, 1960; Ortiz-Santaliestra and Sparling, 2007) and are regulated by endogenous TH level (Shi, 2000; Tata, 2006). Therefore, endocrine disruption might affect energy partitioning at all stages of metamorphosis and tadpoles at different ontogenetic stages might vary in their sensitivity to endocrine disruption due to natural variation in endogenous TH level during development. Previous studies examining the role of THs have focused primarily on metabolic performance, heart rate, respiratory functions or blood pressure during amphibian development (Just *et al.,* 1973; Hou and Burggren, 1995a, 1995b) or have been conducted on a specific ontogenetic stage (Beck and Congdon, 2003; Niehaus *et al.,* 2011; Ruthsatz *et al.,* 2018c, 2019). Few studies have quantified the effect of THs on energetics across life history stages (Pandian and Marian, 1985; Beck and Congdon, 2003; Orlofske and Hopkins, 2009; Orlofske *et al.,* 2017). To the best of our knowledge, only one previous study has quantified the energetic costs of metamorphosis in the laboratory throughout larval development testing the impacts of exogenous stress hormones (Kirschman *et al.,* 2017) and no study to date has investigated physiological ontogeny (i.e. developmental changes in SMR, heart rate and fat reserves) throughout both larval and juvenile stages. Moreover, no study has examined the impact of altered TH caused by environmental stress on ontogenetic changes in physiological traits and how this impact may differ as ontogeny progresses. Given the physiological processes known to be potentially impacted, altered TH levels experienced by early life stages may have serious ‘downstream’ consequences for later life stages and individual fitness (Gomez-Mestre *et al.,* 2010; Burraco *et al.,* 2017; Sinsch *et al.,* 2020; Ruthsatz *et al.,* 2019, 2020).

This study examined developmental changes in SMR, resting heart rate and energy stores and investigated the impact of altered TH levels as caused by environmental stressors on this physiological ontogeny in the common frog (*Rana temporaria*). Endogenous TH levels change naturally throughout amphibian ontogeny with a peak concentration at the onset of metamorphic climax (Shi, 2000; Tata, 2006). Therefore, we hypothesized that the sensitivity of physiological traits to endocrine disruption might differ between ontogenetic stages resulting in life stage-dependent susceptibility to environmental stress. Furthermore, we estimated fitness of juvenile froglets by examining whether an altered TH status experienced during the larval stage affects physiological performance in later life stages. This study provides a framework to better quantify how environmental stressors impact amphibian physiology throughout development that will allow better projections of how stressful environmental conditions will impact both intra- and inter-stage survival and ultimately fitness.

## Material and methods

### Experimental design and animal care

*Rana temporaria* was chosen as the model species because it undergoes a habitat transition after metamorphosis that is associated with complex physiological and morphological changes. Furthermore, it is widely distributed throughout Europe. Five clutches of *R. temporaria* were obtained from Waldpark Marienhöhe in western Hamburg, Germany (53°34′37.4”N 9°46′57.5″E). Eggs hatched and larvae were allowed to develop to stage 25 (free-swimming larvae; Gosner, 1960). From these larvae, 180 individuals originating from different clutches were randomly allocated to the different treatments [L-thyroxine and sodium perchlorate (SP)] and the control group. Fifteen larvae of *R. temporaria* were kept in a standard 9.5-L aquarium filled with 8 L of aged de-chlorinated water (i.e. a total of 12 aquaria: 4 × T4, 4 × SP, 4 × control). The larval density was 1.87 larvae × L^−1^ in the beginning of the experiment. The experiment was conducted in a climate chamber (Weiss Umwelttechnik GmbH, 35 447 Reiskirchen, Germany) with a 12:12 h light:dark cycle at 22 ± 0.1 C.

Larvae were fed 50% high-protein flaked fish food (Sera micron breeding feed for fish and amphibians, Sera, 52 518 Heinsberg, Germany) and 50% spirulina algae. *Ad libitum* rations were provided twice a day to guarantee that food was available in abundance. The size of the rations was continuously adjusted to account for changes in the size of tadpoles and the number of individuals in each aquarium effectively avoiding any restricted feeding conditions that can cause an atrophy of thyroid tissue in a similar manner as TH agonists (Miyata and Ose, 2012). The flakes were free of perchlorate according to the manufacturer. Each day, any dead or abnormal tadpoles were removed from the aquaria (Table A2).

After completing metamorphosis at Gosner stage 46 (Gosner, 1960), all surviving animals were transferred into separate aquaria containing a small amount of water to avoid desiccation, and placed in a climate chamber maintained at 22 ± 0.1 C, representing an average temperature commonly experienced in the field. Froglets were fed *ad libitum* with adult *Drosophila melanogaster* for 7 days prior to being subjected to the final measurements. Exposure to T4 and SP was stopped after completion of metamorphosis. The experiments ran for 7 weeks. All survivors had reached the end of metamorphic climax +7 d at that time (Gosner, 1960; Ortiz-Santaliestra and Sparling, 2007).

### L-thyroxine and SP exposures

We increased internal TH levels (i.e. high TH level) by exposing larvae to 10 μg/L exogenous L-thyroxine (T4, IRMM468 Sigma-Aldrich**,** Sigma-Aldrich, St. Louis, USA), a concentration which is known to influence amphibian metamorphosis (Mann *et al.,* 2009) and is related to increases in T4 observed in larvae responding to stress (Denver, 1997a, 1997b, 1998). Larvae absorb exogenous T4 directly through their permeable skin (Shi, 2000; Tata, 2006; Coady *et al.,* 2010). Exposing larvae to exogenous THs is an established method to simulate the proximate effects of environmental stressors on the TH system (Denver *et al.,* 2002; Tata, 2006; Denver, 2009).

We used a concentration of 250 μg/L SP (99.99% trace metals basis, 381 225 Aldrich, Sigma-Aldrich, St. Louis, USA) to decrease internal TH levels (i.e. low TH level). This concentration of SP is within environmental ranges measured in surface and ground waters of many industrial nations (Motzer, 2001; Tietge *et al.,* 2005; Carr and Theodorakis, 2006; Mukhi and Patiño, 2007) and in bodies of water in which amphibians breed (Ortiz-Santaliestra and Sparling, 2007). SP is an environmentally relevant ED on TH system. As a goitrogen, SP inhibits TH synthesis (Ortiz-Santaliestra and Sparling, 2007), resulting in inhibited amphibian metamorphosis (Tietge *et al.,* 2005).

The T4 and SP treatments were prepared in 0.1 mol/L sodium hydroxide solutions (0.1 N, S2770 SIGMA, Sigma-Aldrich, St. Louis, USA) buffered with 0.1 mol/L muriatic acid solutions as solvents. Solutions were added to the aquaria. To control for any effect of solvents addition, a solution of only 0.1 M sodium hydroxide solution buffered with 0.1 M muriatic acid solution was added to the control aquaria. Water was changed every second day and fresh SP and T4 were added, which is frequent enough to maintain a constant hormone and perchlorate level, in accordance with the standard procedure for chemical and hormonal addition (Miwa and Inui, 1987; Goleman *et al.,* 2002a, 2002b; Iwamuro *et al.,* 2003; Rot-Nikcevic and Wassersug, 2004; Tietge *et al.,* 2005; Ortiz-Santaliestra and Sparling, 2007; Bulaeva *et al.,* 2015).

### Grouping of ontogenetic stages

Ontogenetic stage was determined by evaluating the status of key morphological features typical of specific stages, as detailed in Gosner (1960). Then, each animal was assigned to ontogenetic groups according to the procedure of Ortiz-Santaliestra and Sparling (2007) and Ruthsatz *et al*. (2019) resulting in seven consecutive stage groups: (1) pre-limb (absence of hind limbs; Gosner stages 24–26), (2) limb bud (hind limb visible, but no clear joint formed; Gosner stages 27–34), (3) middle hind limb (knee joint apparent, but toes not completely separated; Gosner stages 35–37), (4) late hind limb (hind limb tubercles and subarticular patches formed; Gosner stages 38–41), (5) onset of metamorphic climax (at least one forelimb present; Gosner stage 42), (6) end of metamorphic climax, (complete resorption of the tail; Gosner stage 46) and (7) juvenile (Gosner stage 46 + 7 days; Ruthsatz *et al.,* 2019). Hereafter, we refer to stage groups 1–5 as tadpoles and to stage groups 6–7 as froglets.

The age was equal to the duration in days after hatching. The snout–vent length (SVL) of the larvae and froglets was measured with a caliper to the nearest 0.5 mm. Specimens were dry blotted and weighed to the nearest 0.001 g with an electronic balance (digital gold scale, Smart Weigh) to measure wet mass (i.e. mass in mg). Hereafter, we refer to ‘wet mass’ as ‘mass’. At the end of the experiment, froglets were euthanized with 200 mg/L of tricaine methanesulfonate (MS-222; Ethyl 3-aminobenzoate methanesulfonate**,** E10521 ALDRICH, Sigma-Aldrich, St. Louis, USA) buffered with 200 mg/L of sodium bicarbonate (S5761 SIGMA, Sigma-Aldrich, St. Louis, USA) (Stuart *et al.,* 2007) and transferred into ethanol (70%) for dissection and measurements of fat content. Survival was expressed as percent (%) of the initial number of individuals at the start of the experiment and determined at each ontogenetic stage (Table A2; Table S8).

### Energy stores

We used different strategies to measure the size of internal energy stores: body condition for whole-body energy stores and the relative size of the fat body, the major energy store tissue in metamorphosed anurans. Body condition was estimated at each of the seven consecutive developmental stages by calculating the scaled mass index (SMI, see below). The fat body was dissected at ontogenetic stage 7 (i.e. juvenile froglet 7 days after completion of metamorphosis).

#### Body condition

The SMI accounts for the allometric relationship between mass and body length and is a standardized measure of the body condition that can be directly compared among individuals (Peig and Green, 2009, 2010; MacCracken and Stebbings, 2012). The SMI has been previously employed as a condition index in anuran larvae (MacCracken and Stebbings, 2012; Dittrich *et al.,* 2016; Ruthsatz *et al.,* 2018b, 2019). A high SMI suggests greater energy stores and, thus, a good body condition. We followed the procedure outlined by Peig and Green (2009) to calculate the SMI for each individual. Slope is calculated from the regression of log transformed SVL and log transformed mass.

$$\begin{align*} &\mathrm{SMI}=\left[\vphantom{\left(\frac{mean\ SVL\ of\ population}{individual\ SVL}\right)} individual\ Mass\right.\\ &\left.\times{\left(\frac{mean\ SVL\ of\ population}{individual\ SVL}\right)}^{slope\ of\ regression\ logMass\sim logSVL}\right] \end{align*}$$

#### Relative fat body size

At stage 7, that fat body was dissected with the aid of a digital stereo-microscope (Keyence VHX-500F). During measurements, froglets or dissected fat bodies were placed in a wax bowl with dark background (Fig. 4A). Fat bodies were dabbed and their mass determined to the nearest 0.01 mg with an electronic balance (Sartorius A200 S). Relative fat body size was calculated from individual mass of fat body (mg) divided by individual body mass (mg) and expressed as mg × $\mathrm{mg}\ {\mathrm{body}\ \mathrm{mass}}^{-1}$.

### Metabolism measurements

Respiration measurements were made on eight randomly chosen individuals from each aquarium at each developmental stage (i.e. 12 aquaria × 8 individuals × 7 stage groups), in total on 672 metabolic rate measurements. Animals at stage groups 1–4 and 7 were not fed 12 h prior to and during the measurement of SMR and were in a post-absorptive state (Orlofske and Hopkins, 2009). No fasting prior to the respiratory measurements was needed at stages 5 and 6 because larvae stop feeding due to the remodelling of mouthparts and digestive tract during metamorphosis and only restart feeding a few days after completion of metamorphosis (Hourdry *et al.,* 1996).

Oxygen consumption was measured by closed respirometry conducted during the natural activity phase between 0900 and 2100 h (Orlofske and Hopkins, 2009). Larvae (stage groups 1–5) were measured in a 30-ml glass respirometer chambers filled with autoclaved tap water to exclude microbial oxygen consumption. Water contained treatment-level concentrations of T4 and SP as experienced by individuals during development. Due to their transition to lung respiration, froglets (stages 6 and 7) were measured in air in 30-ml chambers. Each respirometer was equipped with a fibre optic sensor (Oxygen Dipping Probe DP-PSt7; PreSens Precision Sensing GmbH, Regensburg, Germany) connected to a multichannel oxygen measuring system (Oxy 4 mini; PreSens Precision Sensing GmbH, Regensburg, Germany) and sealed with an air tight rubber plug. The O_2_ concentration was recorded every 15 seconds and measured as ml O_2_$\times{L}^{-1}$ (volume not corrected for the size of the animal). Prior to each trial, the O_2_ fibre optic sensors were calibrated using air-saturated water and a factory-set zero oxygen calibration point at the developmental temperature. Water temperature (22 ± 0.1 C) was controlled by the continuous mixing of the water bath. Oxygen consumption was measured for every animal for 20 min. Empty (control) chambers were run simultaneously in every trial and values were adjusted accordingly. We ensured that less than 10% of total O_2_ was removed during any measurement period to avoid changes in respiration at low O_2_ saturation levels. At the end of the measurements, each animal was removed and its SVL and blotted wet body mass were determined. Tadpoles were placed back into rearing containers. After measurements at stage group 7, froglets were placed back in temperature-controlled containers (in air) temperature-controlled for further experiments.

#### SMR calculations

Prior to statistical analysis, we plotted O_2_ consumption of each animal over time and visually assessed activity peaks to exclude them for the determination of SMR (Orlofske and Hopkins, 2009; Peck and Moyano, 2016; Orlofske *et al.,* 2017). The SMR was expressed in ml O_2_  $\times{h}^{-1}\times{mg}^{-1}$ wet body mass and was determined from the slope of linear least squares regression of O_2_ concentration vs. time (Hastings and Burggren, 1995; Rowe and Funck, 2017; Ruthsatz *et al.,* 2018c, 2019).

#### Heart rate

Because of the developmental change from gill to lung respiration during metamorphosis, we used different experimental techniques for obtaining resting heart rate (f_H_; beats min^−1^) in tadpoles (stage groups 1–5) and froglets (stage groups 6–7). Both tadpoles and froglets were tested at 22 ± 0.1 C water and air temperature, respectively. All measurements were conducted in a temperature-controlled climate chamber.

**Figure 1:**
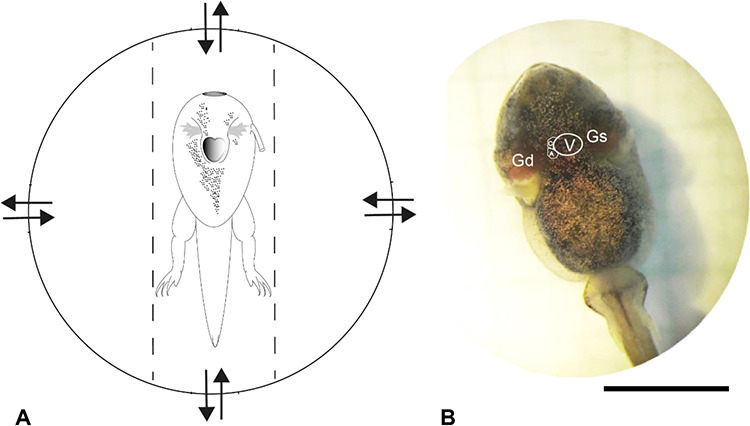
Measurement of resting metabolic heart rate (f_H_) in tadpoles of *R. temporaria*. **A** Fixation chamber made of a Petri dish. Arrows: Vents for continuous water exchange with the surrounding water. Dashed lines: Size-adapted boundaries made of aquarium silicone to maintain tadpole position. Detailed methods in the text. **B** Position of heart in the *R. temporaria* tadpole (stage group 4). V, ventricl*e*. A, auricle. C, *Conus arteriosus*. Gd, right gill. Gs, left gill. At the early stages of development, the tadpoles’ and froglets’ hearts could be readily observed through their low pigmented abdomens. The bar is 5 mm.

Tadpoles were placed individually in fixation chambers made from Petri dishes filled with dechlorinated, aged water (Costa *et al.,* 2008; Fig. 1a). The holding chamber was placed below an analog binocular microscope in a temperature-controlled water bath (± 0.5 C) filled with well-aerated dechlorinated, aged water enriched with respective T4 or SP concentrations. The water bath was equipped with indirect underwater lighting. Four vents in the holding chamber ensured continuous water exchange with the surrounding water within the water bath. Observations were conducted *via* a binocular microscope. At the early stages of development, the tadpoles and froglets and little pigmentation on their abdomens and their heart could be readily observed (Fig. 1b). Froglets were placed individually in 50-ml glass holding chambers filled with 3 mL of dechlorinated, aged water to prevent dehydration of the froglets. Observations were conducted *via* a magnifier at the bottom of the glass beaker. Tadpoles and froglets were allowed 15 min to adjust to the testing environment setting. Typically, tadpoles and froglets rested within a few minutes.

To obtain f_H_, we visually observed the heart and counted the number of beats in 20s for each individual according to the procedure of Niehaus *et al.* (2011) and Watson *et al*. (2014) We measured f_H_ three times for each individual and calculated the mean for our analyses. This procedure provides direct data of the tadpoles’ and froglets’ basal heart rate, since it avoids the stress-induced adrenergic effects due to the implantation of subcutaneous ECG electrodes (e.g. Wassersug *et al.,* 1981; Burggren *et al.,* 1993; Costa *et al.,* 2008).

### Statistical analysis

For all statistical tests R 4.0.2 (R Development Core Team, 2007) for Windows was used. All plots were made using ggplot2 (Wickham, 2009) and Adobe Illustrator CS6. Before the analysis, all independent variables in the models were tested for possible correlations using Spearman’s rank correlation (cor.test function). Consequentially, variables were included in statistical analysis when the correlation was significant but well below the suggested threshold of 0.7 for eliminating variables or particularly needed to be comparable with previous studies (Fielding and Haworth, 1995; Chin, 1998) (Tables S1–S7).

We tested the effect of ‘TH level’ (low TH, control, high TH), ‘ontogenetic stage’ (i.e. stage group 1–7) and the interactive effect of both as fixed factors in separate generalized linear mixed-effect models (GLMMs):glmmPQL for the dependent variables age, SVL, mass, SMR, SMI, f_H_ and relative fat body size [package MASS—generalized linear mixed model with PQL (quasi-likelihood) instead of maximum likelihood (Bolker *et al*., 2008); Table 1]. Although the replicate unit for all GLMMs was the individual, ‘aquarium’ was included as a random factor to address dependencies in the data. *P*-values were obtained from quasi-likelihood-ratio tests (Bolker *et al*., 2008). For relative fat body size, the calculated GLMM was based on ‘TH level’ (control, low TH, high TH) as fixed factor since this variable was measured at only one ontogenetic stage (i.e. stage 7). *N* refers to the total number of analysed individuals, and *n* refers to the total number of tested aquaria.

**Table 1:** Effects of endocrine disruption (i.e. altered TH levels) and ontogenetic stage on age (dah), SVL (mm), body mass (mg), SMR (ml O_2_/mg/h), body condition measured as scaled mass index (SMI), resting heart rate (beats per minute) and relative fat body size (in % body mass) during the development of the common frog (*R. temporaria*). Results were obtained from separate GLMMs, using ‘TH level’ (control, low TH, high TH) and ‘ontogenetic stage’ as well as the interactive effect of both as the fixed factors. The replicate unit for the GLMM analyses was the individual larvae, however to address dependencies in the data (i.e. individuals were raised in the same aquarium), the variable ‘aquarium’ was included as a random factor. For relative fat body size the calculated GLMM was based on ‘TH level’ (control, low TH, high TH) as fixed factor since this variable was measured at only one ontogenetic stage (i.e. stage 7). *N* is the total number of analysed data points, and *n* is the total number of tested aquaria. Significance was set at *P* < 0.05. Low TH level = SP treatment. High TH level = T4 treatment

**GLMM**						
**Dependent variable**	**Fixed effects**	**Estimate**	**SE**	**t-value**	***P***	**N(n)**
Age (dah)	(Intercept)	8.91	0.32	27.75	**<0.001**	1138(12)
	Low TH	3.02	0.45	6.69	**<0.001**	
	High TH	−2.87	0.45	−6.28	**<0.001**	
	Ontogenetic stage	3.20	0.07	42.42	**<0.001**	
	Low TH × ontogenetic stage	0.38	0.11	3.62	**<0.001**	
	High TH × ontogenetic stage	−0.88	0.11	−8.09	**<0.001**	
SVL (mm)	(Intercept)	6.36	0.13	46.53	**<0.001**	1138(12)
	Low TH	0.81	0.19	4.25	**<0.001**	
	High TH	−1.47	0.19	−7.56	**<0.001**	
	Ontogenetic stage	0.80	0.03	24.93	**<0.001**	
	Low TH × ontogenetic stage	0.30	0.04	6.77	**<0.001**	
	High TH × ontogenetic stage	−0.01	0.04	−0.26	0.790	
Mass (mg)	(Intercept)	181.33	11.12	16.31	**<0.001**	1138(12)
	Low TH	−9.66	15.62	−0.62	0.536	
	High TH	−48.92	15.83	−3.09	**0.002**	
	Ontogenetic stage	10.48	2.61	4.02	**<0.001**	
	Low TH × ontogenetic stage	28.65	3.63	7.87	**<0.001**	
	High TH × ontogenetic stage	−9.63	3.77	−2.55	**0.012**	
SMR (ml O_2_/h/mg)	(Intercept)	−0.006	0.001	−4.322	**<0.001**	672(12)
	Low TH	0.002	0.001	0.961	0.337	
	High TH	0.002	0.002	0.303	0.762	
	Ontogenetic stage	0.005	0.001	15.064	**<0.001**	
	Low TH × ontogenetic stage	−0.002	0.002	−4.531	**<0.001**	
	High TH × ontogenetic stage	0.001	0.002	0.274	0.784	
Body condition	(Intercept)	172.20	12.45	13.83	**<0.001**	1138(12)
	Low TH	−0.37	17.49	−0.02	0.983	
	High TH	−37.76	17.73	−2.13	**0.033**	
	Ontogenetic stage	13.29	2.92	4.55	**<0.001**	
	Low TH × ontogenetic stage	27.66	4.07	6.79	**<0.001**	
	High TH × ontogenetic stage	−11.93	4.22	−2.82	**0.004**	
Resting heart rate (bpm)	(Intercept)	17.016	0.730	23.311	**<0.001**	675(12)
	Low TH	−7.761	1.031	−7.527	**<0.001**	
	High TH	36.472	1.032	35.329	**<0.001**	
	Ontogenetic stage	6.339	0.162	38.960	**<0.001**	
	Low TH × ontogenetic stage	−3.167	0.230	−13.744	**<0.001**	
	High TH × ontogenetic stage	−1.092	0.231	−4.736	**<0.001**	
Relative fat body size in % body mass (mg × $\mathrm{mg}\ {\mathrm{body}\ \mathrm{mass}}^{-1}$)	(Intercept)	0.01	0.00	63.38	**<0.001**	119(12)
	Low TH	0.01	0.00	30.20	**<0.001**	
	High TH	−0.01	0.00	−20.65	**<0.001**	

Within each ontogenetic stage, pairwise multiple comparisons between different treatments (low TH, control, high TH), were conducted using Mann–Whitney *U* test with Bonferroni correction (Figs 3, 4B; Table S8).

Descriptive differences in means of dependent variables between Gosner stage groups were expressed as percentage increase or decrease.

**Figure 2:**
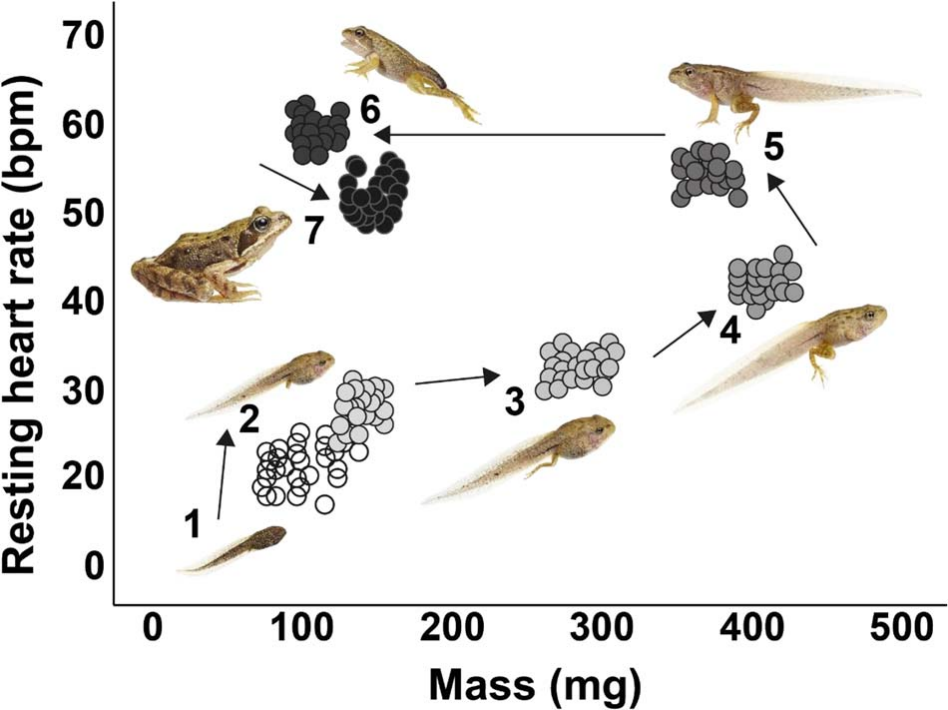
Resting f_H_ plotted as a function of body mass to show simultaneous changes in body mass and mean resting heart rate during development. Correlation analysis revealed that there is a significant relationship between f_H_ and body mass within each of seven developmental groups (see text for further details). Animals were assigned to seven consecutive ontogenetic stage groups according to the procedure of Ortiz-Santaliestra and Sparling (2007) and Ruthsatz *et al*. (2019): Ontogenetic stage groups 1–7: (**1**) pre-limb (absence of hind limbs; Gosner stages 24–26), (**2**) limb bud (hind limb visible, but no clear joint formed; Gosner stages 27–34), (**3**) middle hind limb (knee joint apparent, but toes not completely separated; Gosner stages 35–37), (**4**) late hind limb (hind limb tubercles and subarticular patches formed; Gosner stages 38–41), (**5**) onset of metamorphic climax (at least one forelimb present; Gosner stage 42), (**6**) end of metamorphic climax (complete resorption of the tail; Gosner stage 46) and (**7**) juvenile (Gosner stage 46 + 7 days; Ruthsatz *et al.,* 2019). Photos of *R. temporaria* by Tim Hunt.

A possible correlation between mass and f_H_ was also tested using Spearman’s rank correlation (cor.test function) in control treatment (Fig. 2; Burggren *et al.,* 1992).

## Results

### Energetics during ontogeny

**Figure A1:**
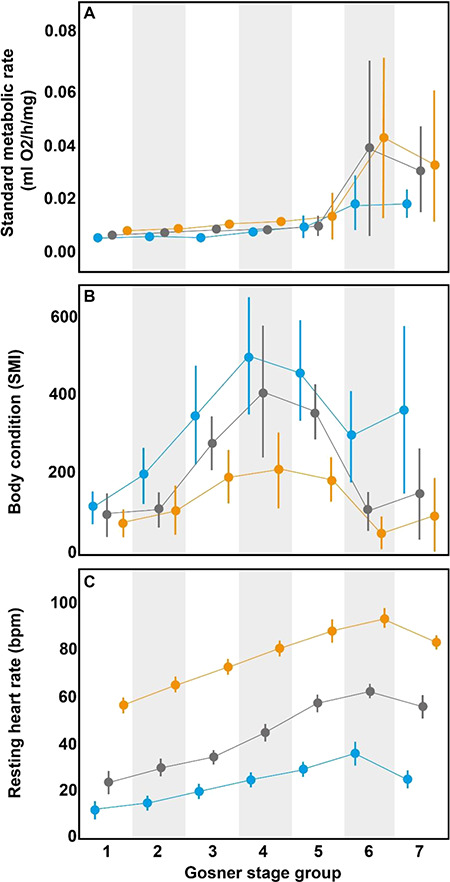
Effects of altered TH levels due to endocrine disruption and ontogenetic stage on mean ± SD **A** standard metabolic rate (mg O_2_/mg/h), **B** body condition measured as scaled mass index (SMI), and **C** resting heart rate (beats per minute) during the development of the common frog (*Rana temporaria*). Blue dots = low TH levels (i.e. SP treatment). Grey dots = Control group. Orange dots = high TH levels (i.e. T4 treatment).

*SMR* –Values of SMR increased in an episodic, non-linear way during ontogeny (R^2^ = 0.49, *P* < 0.001, *N* = 224; Fig. A1). Although the values of SMR were low early in the ontogeny (stage groups 1–3), these values increased from stage groups 3–4 and then again at stage group 5 by approximately 13-fold in total. SMR then decreased by 73% from stage groups 6–7 (i.e. juvenile froglets 7 days after completion of metamorphosis).

*Body condition*—Values of SMI passed through ontogeny in an episodic, non-linear way (R^2^ = 0.04, *P* < 0.001, *N* = 382; Fig. A1). Up to stage group 4, SMI increased. Between stage group 4 and the onset of metamorphosis, SMI and thus, body condition decreased slightly and then decreased 3.8fold during metamorphic climax close to the level of SMI at stage group 1. After completing metamorphosis, SMI increased again until stage group 7.

*Resting heart rate—*f_H_ increased linearly throughout development (R^2^ = 0.86, *P* < 0.001, *N* = 225) until completion of metamorphosis (Fig. A1). Beyond that, f_H_ decreased by 11% until stage group 7. Correlation analysis revealed that there is a significant negative relationship between f_H_ and body mass within each of seven ontogenetic groups (Tables S1–S7, Fig. 2).

### Effects of endocrine disruption on metamorphic traits, survival and energetics

**Figure 3:**
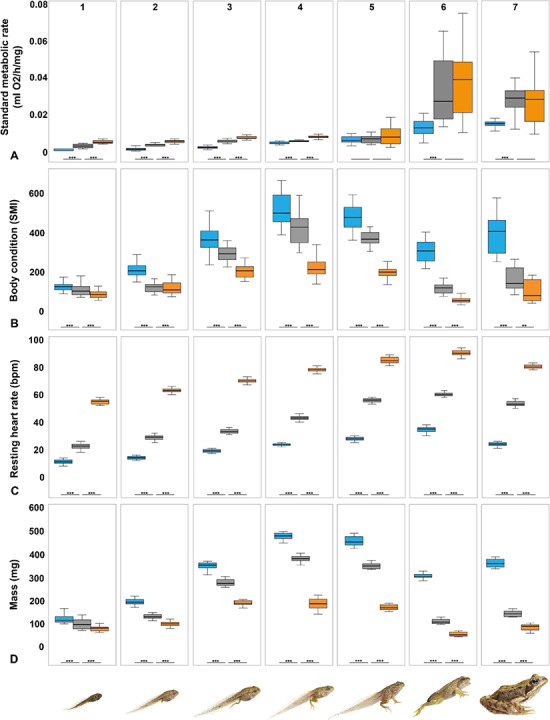
Effects of altered TH levels due to endocrine disruption on metamorphic traits and energetics in larvae and froglets of the common frog (*R. temporaria*) at seven consecutive ontogenetic stages (see text for further details). **A** SMR (ml O_2_/mg/h); **B** Body condition measured as SMI (details in the text); **C** Resting heart rate (beats per minute); and **D** Mass (mg) during the ontogeny of the common frog (*R. temporaria*). Pairwise multiple comparisons were made using Mann–Whitney *U* test with Bonferroni correction. Blue boxes = low TH levels (i.e. SP treatment). Grey boxes = control group. Orange boxes = high TH levels (i.e. T4 treatment). Error bar = median. Box = 1. and 3. quartiles. Dots = outliers, minimum and maximum values. Whiskers = 1.5-fold interquartile range. Asterisks indicate significant differences based on Mann–Whitney *U* tests (^*^P < 0.05; ^**^  *P* < 0.01; ^***^  *P* < 0.001). Photos of *R. temporaria* by Tim Hunt.

Altered TH levels due to endocrine disruption significantly affected metamorphic traits and energetics at most ontogenetic stages and throughout metamorphosis (Table 1; Table S8; Fig. 3). Altered TH levels did not affect the non-linear (i.e. A. SMR and B. SMI) and linear (C. f_H_) episodic way how energetics passed through ontogeny (Fig. A1).

#### Age, size and survival

Age, SVL and mass increased significantly with ontogenetic stage (Table 1). Ontogenetic stage significantly affected the sensitivity of age and mass to altered TH levels (Table 1; Fig. A1). For SVL, ontogenetic stage significantly affected the sensitivity to low but not to high TH levels. Age, SVL and mass were significantly reduced in tadpoles and froglets with high TH levels compared to the control group at all ontogenetic stages showing that high TH levels increase developmental but not growth rate (Table 1; Table S8). Differences in age, mass and SVL between altered TH treatments and control group occurred already at stage 1 and persisted until the completion of metamorphosis. Tadpoles and froglets with low TH levels during development were significantly the oldest and smallest at all stage groups compared to the control group and individuals at high TH levels indicating a reduced developmental and increased growth rate (for mass: Fig. 3D). Survival in all treatments decreased during ontogeny (Table A2) but was lowest in tadpoles and froglets with high TH levels (Table A2; Table S8).

#### Metabolism and energetics

Ontogenetic stage affects the sensitivity of SMR to low but not to high TH levels significantly (Table 1; Fig. A1). SMR increased significantly with ontogenetic stage (Table 1). Altered TH levels significantly reduced SMR at all developmental stages except for stage group 5 (i.e. the onset of metamorphosis) (Fig. 3A; Table S8) but not throughout ontogeny (Table 1). At stage group 1 (i.e. pre-limb tadpole), SMR was significantly reduced in animals at low but not at high TH levels. At stage group 6 (i.e. froglets after completion of metamorphic climax), SMR was significantly reduced in animals at high but not at low TH levels (Fig. 3A). Low TH levels reduced SMR by 67%, 75%, 67%, 18%, 0%, 56% and 43% compared to control group at stage groups 1–7, respectively. In contrast, high TH levels increased SMR by 67%, 50%, 34%, 50%, 14%, 11% and 7% compared to control group at stage group 1–7, respectively.

Ontogenetic stage significantly affected the magnitude of the effect of low TH levels on body condition (i.e. whole-body energy stores; Table 1; Fig. A1). Body condition increased with ontogeny (Table 1). There were differences in body condition between altered TH levels and control group at all ontogenetic stage groups. High TH levels reduced body condition by 20%, 3%, 30%, 48%, 49%, 51% and 35% at stages 1–7, respectively. In contrast, low TH levels increased body condition by 18%, 76%, 25%, 22%, 29%, 177% and 137% at stages 1–7, respectively. Thus, the largest differences between low TH levels and control group were observed between stages 6 and 7, whereas differences between high TH levels and control group were largest directly before and after metamorphic climax.

High TH levels lead to a significant increase in f_H_ at all developmental stages, whereas low TH levels significantly reduced f_H_ (Table 1; Fig. 3C; Table S8). Also, ontogenetic stage had a significant effect on the susceptibility of heart rate to altered TH levels (Table 1; Fig. A1). Low TH levels reduced f_H_ by 50%, 50%, 42%, 44%, 49%, 42% and 55% at stages 1–7, respectively. In contrast, high TH levels increased f_H_ by 145%, 121%, 109%, 79%, 52%, 50% and 51% at stages 1–7, respectively. Thus, the differences between high TH levels and control group in f_H_ were largest at earlier stages, whereas differences between low TH levels were similar at all developmental stages. Differences between altered TH levels and control group were smallest at stage 6 (i.e. froglets after completion of metamorphosis).

#### Fat body in juvenile froglets

**Figure 4:**
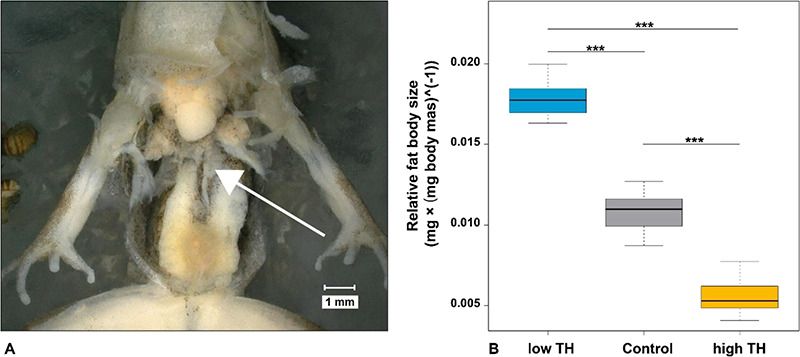
**A** Fat body dissection of a *R. temporaria* froglet from SP treatment (i.e. low TH level) at developmental stage 7 (Gosner stage 46 + 7 days). Arrow: fat body. **B** Effects of TH level on relative fat body size (ratio fat body mass/body mass). Error bar = median. Box = 1. and 3. quartiles. Dots = outliers, minimum and maximum values. Whiskers = 1.5-fold interquartile range. Asterisks indicate significant differences based on Mann–Whitney-U tests (*P < 0.05; ** *P* < 0.01; *** *P* < 0.001).

Size of fat bodies of juvenile froglets at stage 7 was significantly affected by altered TH levels (Table 1; Fig. 4). Low and high TH levels increased and reduced the relative fat body size by 54% and by 46%, respectively (Table S8).

## Discussion

Due to the dramatic changes during ontogeny such as a change from completely aquatic larvae to juveniles that can breathe air and adopt a terrestrial way of life (Burggren *et al.,* 1992), amphibians rely on a fragile balance of energy allocation to growth, development and metabolism (Ruthsatz *et al.,* 2019). Any disturbance to this balance might lead to profound alterations of age, size and energy stores, and thus, might determine survival of amphibians during and across metamorphosis. Here we demonstrate that both morphological and physiological traits are strongly affected by altered TH levels due to endocrine disruption. Further, we demonstrated that the effect of endocrine disruption depends on ontogenetic stage and impacts juvenile performance. Our results allow for more robust projections of how stressful environmental conditions associated with global change may affect across-life stage survival and fitness in the future.

### Changes in metabolism and energy stores during ontogeny of *R. temporaria*

Illustrating the standard transition of metamorphic and physiological traits throughout ontogeny is fundamental to understanding environmental impacts since development *per se* may modify body functions (Pelster, 1999). In this study, resting heart rate was negatively related to mass within each of seven ontogenetic groups, but increased linearly with developmental stage until completion of metamorphosis. Given these stage-specific relationships, we suggest that body mass does not explain all of the changes observed in resting heart rate. An increase in resting heart rate in the present study could rather be explained by an increased demand in the circulatory system due to a switch from cutaneous and gill respiration to cutaneous and lung respiration as development proceeds (Burggren and West, 1982; Burggren and Doyle, 1986; Taylor *et al.,* 2014). In contrast, Burggren and Doyle (1986) and Hou and Burggren (1995b) found a general decrease in resting heart rate during ontogeny in tadpoles of both the bullfrog (*R. catesbeiana*) and *Xenopus laevis*. A decline in heart rate with larval or foetal ontogeny has also been recorded in other vertebrates including fish, frogs, and birds, but there are many exceptions to this pattern (reviewed in Hou and Burggren, 1995a; Burggren and Warburton, 1994, and Pelster, 1999). Consequently, there appears to be no generalized pattern of heart rate change in early ontogeny among anuran amphibians.

Since amphibian larvae occupy an aquatic environment, key changes in respiratory and metabolic function may be expected to occur during the transition from water to air breathing (Just *et al.,* 1973). Thus, it would appear that, during the stages with highest SMR and resting heart rate, these animals possess three respiratory organs: gills, lungs and skin, with lungs assuming an increasing importance in gas exchange (Just *et al.,* 1973; Pelster, 1999). Indeed, SMR and resting heart were highest in late larval stages prior to the onset of metamorphic climax. Moreover, both rates were relatively low during early development when tadpoles rely on cutaneous diffusion and gill breathing. In vertebrates, heart and mass-specific metabolic rate tend to decrease with increasing body mass (Kleiber, 1947, 1961; Lillywhite *et al.,* 1999; Pelster, 1999; Hulbert and Else, 2004), although isometric scaling (no decrease or increase with increasing mass) has been reported during the early larval period in various species of fish (see review by Peck and Moyano, 2016). Here, we confirm this pattern of decline with increasing mass for heart rate within each developmental stage.

Accumulating large energy stores before the onset of metamorphic climax is important for successfully fueling growth, development and metabolism throughout ontogeny but particularly during metamorphic climax (Orlofske and Hopkins, 2009). Indeed, larvae of *R. temporaria* did not only increase their body mass but also the size of internal energy stores before the onset of metamorphosis (stage group 5). Both body mass and size of energy stores were lowest after completion of metamorphosis indicating the use of internal energy stores to fuel energetic demands during metamorphic climax and increased immediately after froglets of *R. temporaria* started feeding.

### Metabolism and energy stores were affected by altered TH levels throughout metamorphosis but ontogenetic stage modulates the sensitivity of *R. temporaria* to endocrine disruption

Endocrine disruption of the TH system led to differences in resting heart rate, SMR and accumulation of internal energy stores due to the metabolic function of THs throughout ontogeny. This is in accordance with results of earlier studies evaluating THs as determinant on metabolic processes in vertebrates (e.g. Steyermark *et al*., 2005; Ruthsatz *et al.,* 2018c; McNabb and King, 1993; Oommen and Sreejith, 2006). However, our results also demonstrate that the ontogenetic stage modulates the sensitivity of *R. temporaria* to effects of endocrine disruption: Differences between the treatments already occurred during the first stage of larval development examined here (i.e. stage group 1) indicating that altered TH levels have immediate effects that last until completion of metamorphosis. It is therefore likely that environmental stress associated with endocrine disruption would affect tadpoles in their natural habitat directly after hatching. However, the impact of endocrine disruption on metabolism and energy stores was lowest 7 days after completion of metamorphosis or 7 days post-exposure, indicative of a compensatory response in juveniles once removed from the endocrine stressor. Consequently, if juvenile froglets disperse from their larval habitat after completing metamorphosis, the effects of the previous exposure to stress may be alleviated. This is in line with the occurrence of growth compensation after animals were removed from exposure to larval stressors (e.g. Capellán and Nicieza, 2007; Squires *et al.,* 2010; Hector *et al.,* 2012; Dahl *et al.,* 2012).

All aspects of fat metabolism including synthesis, mobilization and degradation are induced by THs in vertebrates (Pucci *et al.,* 2000). Thus, endocrine disruption of TH level might affect the capacity to store fats in internal storages. In the present study, size of internal energy stores was affected by altered TH levels in tadpoles and post-metamorphic froglets of *R. temporaria.* On the one hand, this impact on energy stores is due to the increasing effect of THs on basal energy expenditure by increased activities of enzymes and densities of mitochondria in metabolic relevant tissues such as liver and red skeletal muscle with indirect consequences for the fat metabolism (Ruthsatz *et al.,* 2018b; Pucci *et al.,* 2000; Rowe *et al.,* 1998; Beck and Congdon, 2003). On the other hand, THs modify the activity of other regulatory hormones such as insulin, glucagon and catecholamines with direct consequences for fat metabolism (Pucci *et al.,* 2000). Indeed, we could demonstrate an effect of TH level on SMR in tadpoles and froglets of *R. temporaria*. This effect, however, was not consistent throughout ontogeny, since there was no effect on SMR at the onset of metamorphic climax. We suggest that SMR is not only determined by TH level but also by other mechanisms such as a response to the altered resting heart rates or by variation in the levels of corticosterone (Kirschman *et al.,* 2017; MacDougall-Shackleton *et al.,* 2019), which can synergize with TH (Denver, 2009) and are known to increase during development and due to stress exposure (Dantzer *et al.,* 2014; Burraco and Gomez-Mestre, 2016; reviewed in Hammond *et al.,* 2020).

THs determine not only SMR but also resting heart rate (Dillmann, 2002; Little and Seebacher, 2014a, 2014b). In the present study, effects of altered TH levels on resting heart rate were immense. Individuals exposed to high TH levels had resting heart rates that were, on average, 86% higher than those in the control treatment whereas low TH levels decreased resting heart rate by an average of 47%. Both, hyperthyroidism and hypothyroidism (i.e. increased and decreased TH levels, respectively) are known to increase the risk for heart failure in vertebrates (Schmidt-Ott and Ascheim, 2006). Consequently, endocrine disruption by environmental stress and aquatic contaminants might result in carry-over effects on later life stages by causing TH-induced heart failure in juveniles and adults. Here, we also observed mortality of individuals exposed to high TH levels. In addition, further stressors such as thermal stress from heat waves and diseases such as the amphibian pathogen *Batrachochytrium dendrobatidis* are also known to increase the risk of heart failure which would be exacerbated in individuals suffering from altered TH levels (Pelster, 1999; Voyles *et al.,* 2009; Salla *et al.,* 2015).

Here, carry-over effects of altered TH levels during metamorphosis were investigated by measuring juvenile physiological performance in terms of the relative size of the fat body, which is a proxy for post-metamorphic size of energy stores and metabolic health (Wright *et al.,* 2011). We found that high TH levels dramatically decreased the size of fat bodies. In temperate anurans such as the common frog (*R. temporaria*), energy stores are built after emergence before the onset of the next winter (reviewed by Reading and Clarke, 1995; Chen *et al*., 2011). Consequently, froglets from a stressful larval habitat and emerging with less body fat will need to rapidly replenish and increase their energy stores. A previous study reported that the locomotor performance of juveniles was impaired by high TH levels due to the smaller body size at emergence from metamorphosis (Ruthsatz *et al.,* 2019). Strong locomotor performance is also extremely beneficial for accumulating energy storages needed for a variety of processes such as gonad development and survival during hibernation (Reading and Clarke, 1995). In addition, altered TH levels might also reduce locomotor performance through carry-over effects of altered developmental and growth rates (Gomez-Mestre *et al.,* 2010; Burraco *et al.,* 2017; Sinsch *et al.,* 2020). Therefore, froglets which experienced environmental stress during the larval stage may suffer in three different ways: (1) a smaller fat body size at emergence, (2) a small body size which reduces locomotor performance and in turn impedes foraging and thus, (3) lesser storage of energy reserves essential for successful overwintering.

## Conclusions

This study offers a physiological-based understanding of how environmental stressors can dramatically disturb the balance among growth, development, cardiac activity and energy storage during amphibian ontogeny by endocrine disruption. Even if juveniles disperse from their larval habitat, they might suffer from direct and indirect carry-over effects of endocrine disruption experienced during metamorphosis (Ruthsatz *et al.,* 2019; Yagi and Green, 2018; Bouchard *et al.,* 2016; Sinsch *et al.,* 2020). Moreover, metamorphosis is not a new beginning (Pechenik *et al.,* 1998), since migration capacity in amphibians is often limited (Yu *et al.,* 2013; Sinsch, 2014) and metamorphs might also experience stressful environmental conditions in terrestrial habitats continuing endocrine disruption of TH and other hormone systems. Crespi and Warne (2013) could demonstrate that the environmental conditions experienced during the larval stage alter the hormonal stress response in juveniles indicating a carry-over effect of endocrine disruption on the physiological stress response level. Long-term studies are needed to understand the downstream consequences of altered TH levels as caused by environmental stressors during larval and early juvenile stages on the phenotype and fitness of the adults. By doing so, investigating different biomarkers of physiological stress responses to sub-optimal conditions associated with global change might be a helpful tool in amphibian conservation physiology (Dantzer *et al.,* 2014; Walls and Gabor, 2019). Results of those studies will enable us to more holistically understand stress responses to changing environmental conditions and how sublethal stress can interact with ecological and evolutionary processes to contribute to the observed declines in amphibian populations and aid in the development of conservation strategies.

## Author contributions

K.R., K.D., M.P. and J.G. conceived and designed the study. K.R. and N.S. conducted the experiments. K.R., K.P. and P.B. performed the statistical analysis. All authors participated in manuscript editing and final approval.

## Conflict of Interest

None declared.

## Statement of Ethics

The authors have no ethical conflicts to disclose. The experiments were conducted under permission from the *Amt für Verbraucherschutz, Lebensmittelsicherheit und Veterinärwesen* in Hamburg, Germany (Billstraße 80, D- 20539 Hamburg; Gz. V1305/591-00.33, Nr. 03/16). The permission for extraction of spawn of the common frog (*R. temporaria*) was granted by the *Amt für Naturschutz, Grünplanung und Energie* (Neuenfelder Straße 19, D-21109 Hamburg; Gz. NGE3102/897.20-90).

## Funding

This research did not receive any specific grant from funding agencies in the public, commercial or not-for-profit sectors.

## Supplementary Material

coaa100_Manuscript_revised_nocorrections

## Appendix 1

**Table A1:** Effects of altered TH levels on means (±SD) of metamorphic traits and energetics in larvae and froglets of the common frog (*R. temporaria*) at consecutive ontogenetic stages (see text for further details). N is the total number of analysed individual animals. Low TH level = SP treatment. High TH level = T4 treatment. N for individuals measured. Four replicates (i.e. aquaria) per treatment

**Developmental stage**	**Metamorphic traits and energetics**	**Mean (SD)**					
		**Low TH**	**N**	**Control**	**N**	**High TH**	**N**
**1** (Gosner stages 24 to 26)	Age (dah)	18.11 (0.74)	60	13.66 (1.45)	60	8.94 (1.11)	60
	Snout-vent length (mm)	7.82 (0.68)	60	6.77 (0.96)	60	5.42 (0.67)	60
	Mass (mg)	117.58 (15.87)	60	97.02 (19.43)	60	78.48 (10.54)	60
	Standard metabolic rate (ml O_2_/h/mg)	0.0011 (0.0003)	32	0.0039 (0.0008)	32	0.0062 (0.0009)	32
	Body condition	118.47 (20.41)	60	100.08 (27.11)	60	80.11 (18.43)	60
	Resting heart rate (bpm)	11.16 (1.48)	32	22.34 (2.28)	32	54.69 (1.69)	32
**2** (Gosner stages 27 to 34)	Age (dah)	19.46 (1.24)	60	16.00 (1.03)	59	10.45 (0.98)	59
	Snout-vent length (mm)	8.95 (0.97)	60	7.56 (1.14)	59	6.55 (1.11)	59
	Mass (mg)	197.67 (13.05)	60	136.73 (8.86)	59	105.75 (10.78)	59
	Standard metabolic rate (ml O_2_/h/mg)	0.0012 (0.0004)	32	0.0048 (0.0007)	32	0.0068 (0.0007)	32
	Body condition	201.82 (35.13)	60	114.59 (22.10)	59	111.29 (30.61)	59
	Resting heart rate (bpm)	13.94 (1.12)	32	28.75 (1.83)	32	62.81 (1.69)	32
**3** (Gosner stages 35 to 37)	Age (dah)	21.17 (3.48)	60	18.05 (1.93)	59	12.89 (0.94)	58
	Snout-vent length (mm)	10.24 (1.44)	60	9.14 (0.79)	59	7.21 (0.99)	58
	Mass (mg)	347.08 (16.92)	60	281.61 (13.17)	59	194.38 (10.25)	58
	Standard metabolic rate (ml O_2_/h/mg)	0.0021 (0.0003)	32	0.0063 (0.0006)	32	0.0083 (0.0007)	32
	Body condition	356.51 (63.34)	60	284.65 (35.37)	59	199.14 (34.51)	58
	Resting heart rate (bpm)	18.66 (1.23)	32	33.37 (1.43)	32	70.33 (1.49)	32
**4** (Gosner stages 38 to 41)	Age (dah)	24.64 (1.68)	59	19.68 (1.058)	57	15.93 (1.59)	55
	Snout-vent length (mm)	13.24 (1.05)	59	10.61 (1.11)	57	8.77 (0.89)	55
	Mass (mg)	13.23 (1.05)	59	401.54 (11.82)	57	213.24 (24.74)	55
	Standard metabolic rate (ml O_2_/h/mg)	499.41 (26.46)	32	0.0062 (0.0005)	32		32
	Body condition	0.0051 (0.0005)	59	413.77 (85.03)	57	215.58 (48.46)	55
	Resting heart rate (bpm)	506.59 (74.94)	32	43.06 (1.36)	32	77.69 (1.53)	32
**5** (Gosner stage 42)	Age (dah)	26.08 (3.21)	59	20.96 (2.04)	55	16.06 (2.30)	49
	Snout-vent length (mm)	13.49 (0.97)	59	10.50 (0.51)	55	8.52 (0.67)	49
	Mass (mg)	462.46 (19.24)	59	362.00 (11.51)	55	188.80 (11.40)	49
	Standard metabolic rate (ml O_2_/h/mg)	0.0075 (0.0019)	32	0.0075 (0.0021)	32	0.0091 (0.0045)	32
	Body condition	468.51 (63.03)	59	364.32 (35.33)	55	190.68 (27.59)	49
	Resting heart rate (bpm)	28.06 (1.36)	32	55.88 (1.52)	32	85.00 (2.27)	32
**6** (Gosner stage 46)	Age (dah)	34.34 (2.79)	55	28.58 (2.13)	53	18.19 (1.83)	42
	Snout-vent length (mm)	13.52 (1.13)	55	10.82 (1.09)	53	9.51 (1.92)	42
	Mass (mg)	293.69 (10.74)	55	105.21 (9.68)	53	52.26 (8.39)	42
	Standard metabolic rate (ml O_2_/h/mg)	0.0162 (0.0051)	32	0.0365 (0.0164)	32	0.0407 (0.0171)	32
	Body condition	300.88 (57.35)	55	108.83 (52.09)	53	53.46 (20.52)	42
	Resting heart rate (bpm)	34.47 (2.14)	32	60.09 (1.27)	32	90.38 (1.91)	32
**7** (Gosner stage 46 + 7 days)	Age (dah)	40.91 (2.77)	46	35.82 (2.23)	39	25.17 (1.83)	34
	Snout-vent length (mm)	13.69 (1.05)	46	11.41 (1.14)	39	10.26 (1.23)	34
	Mass (mg)	353.04 (15.92)	46	143.13 (11.86)	39	87.82 (13.18)	34
	Standard metabolic rate (ml O_2_/h/mg)	0.0158 (0.0026)	32	0.0285 (0.0081)	32	0.0306 (0.0169)	32
	Body condition	370.93 (106.64)	46	156.13 (59.11)	39	99.86 (47.70)	34
	Resting heart rate (bpm)	23.91 (1.35)	32	53.36 (2.10)	32	80.41 (1.45)	32
	Relative fat body size in % body mass (mg × $\mathrm{mg}\ {\mathrm{body}\ \mathrm{mass}}^{-1}$)	0.017 (0.002)	46	0.011 (0.001)	39	0.005 (0.002)	34
**7** (Gosner stage 46 + 7 days)	Age (dah)	40.91 (2.77)	46	35.82 (2.23)	39	25.17 (1.83)	34
	Snout-vent length (mm)	13.69 (1.05)	46	11.41 (1.14)	39	10.26 (1.23)	34
	Mass (mg)	353.04 (15.92)	46	143.13 (11.86)	39	87.82 (13.18)	34
	Standard metabolic rate (ml O_2_/h/mg)	0.0158 (0.0026)	32	0.0285 (0.0081)	32	0.0306 (0.0169)	32
	Body condition	370.93 (106.64)	46	156.13 (59.11)	39	99.86 (47.70)	34
	Resting heart rate (bpm)	23.91 (1.35)	32	53.36 (2.10)	32	80.41 (1.45)	32
	Relative fat body size in % body mass (mg × $\mathrm{mg}\ {\mathrm{body}\ \mathrm{mass}}^{-1}$)	0.017 (0.002)	46	0.011 (0.001)	39	0.005 (0.002)	34

**Table A2:** Effects of altered TH levels on mean (±SD) survival (%) in larvae and froglets of the common frog (*R. temporaria*) at consecutive ontogenetic stages (see text for further details). Low TH level = SP treatment. High TH level = T4 treatment. N for individuals measured. n is the total number of tested aquaria

**Developmental stage**	**Mean (SD)**					
	**Low TH**	**N(n)**	**Control**	**N(n)**	**High TH**	**N(n)**
**1** (Gosner stages 24 to 26)	100	60(4)	100	60(4)	100	60(4)
**2** (Gosner stages 27 to 34)	100	60(4)	98.33 (3.33)	59(4)	98.33 (3.33)	59(4)
**3** (Gosner stages 35 to 37)	100	60(4)	98.33 (3.33)	59(4)	96.66 (3.84)	58(4)
**4** (Gosner stages 38 to 41)	98.33 (3.33)	59(4)	95.00 (3.33)	57(4)	91.66 (6.38)	55(4)
**5** (Gosner stage 42)	98.33 (3.33)	59(4)	93.21 (0.28)	55(4)	83.21 (8.42)	49(4)
**6** (Gosner stage 46)	91.66 (3.33)	55(4)	89.76 (4.41)	53(4)	72.61 (6.87)	42(4)
**7** (Gosner stage 46 + 7 days)	78.09 (5.97)	46(4)	66.66 (3.36)	38(4)	62.09 (6.65)	34(4)
