# Supplementary material for: Shifts in sensitivity of amphibian metamorphosis to endocrine disruption: the common frog (Rana temporaria) as a case study
Source: Conserv Physiol. 2020 Dec 14;8(1):coaa100. doi: 10.1093/conphys/coaa100 (PMC7735370; doi:10.1093/conphys/coaa100)
Supplement: coaa100_Manuscript_revised_nocorrections [file coaa100_manuscript_revised_nocorrections.docx]

**Supplementary material**

**Table S1.** Spearman’s rank correlation of dependent variables at developmental stage 1 (i.e., pre-limb tadpole). N = 180 (N = 96 for f_H_). Regular: Coefficient of correlation (ρ). Italic: P- values. Bold: Significant high correlations.

|  | Mass (mg) | Age (dah) | SVL (mm) | f_H_ (bpm) |
| --- | --- | --- | --- | --- |
| Mass (mg) | - | 0.693 | 0.516 | -0.651 |
| Age (dah) | *<0.001* | - | **0.748** | **-0.904** |
| SVL (mm) | *<0.001* | *<0.001* | - | **-0.776** |
| f_H_ (bpm) | *<0.001* | *<0.001* | *<0.001* | - |

**Table S2.** Spearman’s rank correlation of dependent variables at developmental stage 2 (i.e., limb bud tadpole). N = 178 (N = 96 for f_H_). Regular: Coefficient of correlation (ρ). Italic: P- values. Bold: Significant high correlations.

|  | Mass (mg) | Age (dah) | SVL (mm) | f_H_ (bpm) |
| --- | --- | --- | --- | --- |
| Mass (mg) | - | **0.886** | 0.629 | **-0.867** |
| Age (dah) | *<0.001* | - | **0.782** | **-0.892** |
| SVL (mm) | *<0.001* | *<0.001* | - | -0.582 |
| f_H_ (bpm) | *<0.001* | *<0.001* | *<0.001* | - |

**Table S3.** Spearman’s rank correlation of dependent variables at developmental stage 3 (i.e., middle hind limb tadpole). N = 177 (N = 96 for f_H_). Regular: Coefficient of correlation (ρ). Italic: P- values. Bold: Significant high correlations.

|  | Mass (mg) | Age (dah) | SVL (mm) | f_H_ (bpm) |
| --- | --- | --- | --- | --- |
| Mass (mg) | - | **0.771** | **0.702** | **-0.880** |
| Age (dah) | *<0.001* | - | 0.658 | **-0.775** |
| SVL (mm) | *<0.001* | *<0.001* | - | **-0.705** |
| f_H_ (bpm) | *<0.001* | *<0.001* | *<0.001* | - |

**Table S4.** Spearman’s rank correlation of dependent variables at developmental stage 4 (i.e., late hind limb tadpole). N = 171 (N = 96 for f_H_). Regular: Coefficient of correlation (ρ). Italic: P- values. Bold: Significant high correlations.

|  | Mass (mg) | Age (dah) | SVL (mm) | f_H_ (bpm) |
| --- | --- | --- | --- | --- |
| Mass (mg) | - | **0.885** | **0.839** | **-0.913** |
| Age (dah) | *<0.001* | - | **0.824** | **-0.871** |
| SVL (mm) | *<0.001* | *<0.001* | - | **-0.819** |
| f_H_ (bpm) | *<0.001* | *<0.001* | *<0.001* | - |

**Table S5.** Spearman’s rank correlation of dependent variables at developmental stage 5 (i.e., at the onset of metamorphosis). N = 163 (N = 96 for f_H_). Regular: Coefficient of correlation (ρ). Italic: P- values. Bold: Significant high correlations.

|  | Mass (mg) | Age (dah) | SVL (mm) | f_H_ (bpm) |
| --- | --- | --- | --- | --- |
| Mass (mg) | - | **0.823** | **0.895** | **-0.903** |
| Age (dah) | *<0.001* | - | **0.782** | **-0.834** |
| SVL (mm) | *<0.001* | *<0.001* | - | **-0.885** |
| f_H_ (bpm) | *<0.001* | *<0.001* | *<0.001* | - |

**Table S6.** Spearman’s rank correlation of dependent variables at developmental stage 6 (i.e., froglet after completion of metamorphosis). N = 150 (N = 96 for f_H_). Regular: Coefficient of correlation (ρ). Italic: P- values. Bold: Significant high correlations.

|  | Mass (mg) | Age (dah) | SVL (mm) | f_H_ (bpm) |
| --- | --- | --- | --- | --- |
| Mass (mg) | - | **0.875** | **0.766** | **-0.892** |
| Age (dah) | *<0.001* | - | **0.718** | **-0.868** |
| SVL (mm) | *<0.001* | *<0.001* | - | **-0.766** |
| f_H_ (bpm) | *<0.001* | *<0.001* | *<0.001* | - |

**Table S7.** Spearman’s rank correlation of dependent variables at developmental stage 7 (i.e., juvenile froglet 7 days after completion of metamorphosis). N = 119 (N = 96 for f_H_). Regular: Coefficient of correlation (ρ). Italic: P- values. Bold: Significant high correlations.

|  | Mass (mg) | Age (dah) | SVL (mm) | f_H_ (bpm) |
| --- | --- | --- | --- | --- |
| Mass (mg) | - | **0.812** | **0.762** | **-0.895** |
| Age (dah) | *<0.001* | - | **0.756** | **-0.873** |
| SVL (mm) | *<0.001* | *<0.001* | - | **-0.729** |
| f_H_ (bpm) | *<0.001* | *<0.001* | *<0.001* | - |

**Table S8.** Effects of altered TH levels due to endocrine disruption on metamorphic traits and energetics in larvae and froglets of the common frog (*R. temporaria*) at consecutive ontogenetic stages (see text for further details). N is the total number of analyzed individual animals. Pairwise multiple comparisons were made using Mann-Whitney-U test with Bonferroni correction. Significance was set at P < 0.05. Low TH level = SP treatment. High TH level = T4 treatment.

| **Developmental stage** | **Dependent variable** | **Mann-Whitney-U test (pairwise comparisons)** | | | | | | | | | | | |
| --- | --- | --- | --- | --- | --- | --- | --- | --- | --- | --- | --- | --- | --- |
|  |  | **Control - SP** | | | | **Control - T4** | | | | **SP - T4** | | | |
|  |  | **U** | **z** | **P** | **N** | **U** | **z** | **P** | **N** | **U** | **z** | **P** | **N** |
| **1** | Snout-vent length (mm) | 12.00 | -9.45 | **<0.001** | 120 | 6.00 | -9.48 | **<0.001** | 120 | 0.00 | -9.52 | **<0.001** | 120 |
|  | Mass (mg) | 757.50 | -5.56 | **<0.001** | 120 | 526.50 | -6.76 | **<0.001** | 120 | 7.00 | -9.348 | **<0.001** | 120 |
|  | Age (dah) | 817.50 | -5.16 | **<0.001** | 120 | 898.50 | -4.74 | **<0.001** | 120 | 12.00 | -9.39 | **<0.001** | 120 |
|  | Standard metabolic rate (ml O_2_/h/mg) | 0.00 | -7.23 | **<0.001** | 64 | 44.00 | -6.43 | **<0.001** | 64 | 0.00 | -7.23 | **<0.001** | 64 |
|  | Body condition | 1013.00 | -4.13 | **<0.001** | 120 | 1015.00 | -4.12 | **<0.001** | 120 | 302.00 | -7.86 | **<0.001** | 120 |
|  | Resting heart rate (bpm) | 0.00 | -6.90 | **<0.001** | 64 | 0.00 | -6.89 | **<0.001** | 64 | 0.00 | -6.91 | **<0.001** | 64 |
|  | Survival (%) | 8.00 | 0.00 | 1.000 | NA | 8.00 | 0.00 | 1.000 | NA | 8.00 | 0.00 | 1.000 | NA |
| **2** | Snout-vent length (mm) | 91.50 | -9.05 | **<0.001** | 119 | 0.00 | -9.48 | **<0.001** | 118 | 0.00 | -9.51 | **<0.001** | 119 |
|  | Mass (mg) | 647.50 | -6.01 | **<0.001** | 119 | 1072.00 | -3.63 | **<0.001** | 118 | 199.50 | -8.42 | **<0.001** | 119 |
|  | Age (dah) | 0.00 | -9.42 | **<0.001** | 119 | 31.00 | -9.21 | **<0.001** | 118 | 0.00 | -9.42 | **<0.001** | 119 |
|  | Standard metabolic rate (ml O_2_/h/mg) | 0.00 | -7.15 | **<0.001** | 64 | 42.00 | -6.48 | **<0.001** | 64 | 0.00 | -7.18 | **<0.001** | 64 |
|  | Body condition | 25.00 | -9.25 | **<0.001** | 119 | 1553.00 | -1.01 | 0.313 | 118 | 101.00 | -8.87 | **<0.001** | 119 |
|  | Resting heart rate (bpm) | 0.00 | -6.97 | **<0.001** | 64 | 0.00 | -6.90 | **<0.001** | 64 | 0.00 | -6.97 | **<0.001** | 64 |
|  | Survival (%) | 6.00 | -1.00 | 0.317 | NA | 8.00 | 0.00 | 1.00 | NA | 6.00 | -1.00 | 0.317 | NA |
| **3** | Snout-vent length (mm) | 719.00 | -5.63 | **<0.001** | 119 | 0.00 | -9.42 | **<0.001** | 117 | 58.00 | -9.14 | **<0.001** | 118 |
|  | Mass (mg) | 947.00 | -4.42 | **<0.001** | 119 | 287.00 | -7.84 | **<0.001** | 117 | 112.00 | -8.82 | **<0.001** | 118 |
|  | Age (dah) | 0.00 | -9.41 | **<0.001** | 119 | 0.00 | -9.33 | **<0.001** | 117 | 0.00 | -9.37 | **<0.001** | 118 |
|  | Standard metabolic rate (ml O_2_/h/mg) | 0.00 | -7.32 | **<0.001** | 64 | 20.50 | -6.84 | **<0.001** | 64 | 0.00 | -7.29 | **<0.001** | 64 |
|  | Body condition | 596.00 | -6.24 | **<0.001** | 119 | 128.00 | -8.63 | **<0.001** | 117 | 53.00 | -9.08 | **<0.001** | 118 |
|  | Resting heart rate (bpm) | 0.00 | -6.91 | **<0.001** | 64 | 0.00 | -6.96 | **<0.001** | 64 | 0.00 | -6.97 | **<0.001** | 64 |
|  | Survival (%) | 6.00 | -1.00 | 0.317 | NA | 6.00 | -0.68 | 0.495 | NA | 4.00 | -1.53 | 0.127 | NA |
| **4** | Snout-vent length (mm) | 28.00 | -9.21 | **<0.001** | 116 | 65.00 | -8.85 | **<0.001** | 112 | 0.00 | -9.25 | **<0.001** | 114 |
|  | Mass (mg) | 150.00 | -8.55 | **<0.001** | 116 | 315.00 | -7.35 | **<0.001** | 112 | 0.00 | -9.28 | **<0.001** | 114 |
|  | Age (dah) | 0.00 | -9.29 | **<0.001** | 116 | 0.00 | -9.13 | **<0.001** | 112 | 0.00 | -9.21 | **<0.001** | 144 |
|  | Standard metabolic rate (ml O_2_/h/mg) | 102.00 | -5.92 | **<0.001** | 64 | 0.00 | -7.51 | **<0.001** | 64 | 0.00 | -7.55 | **<0.001** | 64 |
|  | Body condition | 743.00 | -5.18 | **<0.001** | 116 | 42.00 | -8.88 | **<0.001** | 112 | 0.00 | -9.20 | **<0.001** | 114 |
|  | Resting heart rate (bpm) | 0.00 | -6.95 | **<0.001** | 64 | 0.00 | -6.92 | **<0.001** | 64 | 0.00 | -6.94 | **<0.001** | 64 |
|  | Survival (%) | 4.00 | -1.32 | 0.186 | NA | 5.00 | -0.94 | 0.350 | NA | 3.00 | -1.56 | 0.119 | NA |
| **5** | Snout-vent length (mm) | 305.00 | -7.50 | **<0.001** | 114 | 176.00 | -7.66 | **<0.001** | 104 | 3.50 | -8.92 | **<0.001** | 108 |
|  | Mass (mg) | 0.00 | -9.29 | **<0.001** | 114 | 23.00 | -8.73 | **<0.001** | 104 | 0.00 | -8.98 | **<0.001** | 108 |
|  | Age (dah) | 0.00 | -9.21 | **<0.001** | 114 | 0.00 | -8.78 | **<0.001** | 104 | 0.00 | -8.93 | **<0.001** | 108 |
|  | Standard metabolic rate (ml O_2_/h/mg) | 508.00 | -0.05 | 0.957 | 64 | 451.00 | -0.82 | 0.413 | 64 | 441.00 | -0.95 | 0.340 | 64 |
|  | Body condition | 260.00 | -7.73 | **<0.001** | 114 | 0.00 | -8.77 | **<0.001** | 104 | 0.00 | -8.92 | **<0.001** | 108 |
|  | Resting heart rate (bpm) | 0.00 | -6.91 | **<0.001** | 64 | 0.00 | -6.91 | **<0.001** | 64 | 0.00 | -6.90 | **<0.001** | 64 |
|  | Survival (%) | 1.50 | -2.05 | **0.040** | NA | 0.50 | -2.23 | **0.026** | NA | 0.00 | -2.36 | **0.018** | NA |
| **6** | Snout-vent length (mm) | 165.00 | -7.98 | **<0.001** | 108 | 0.00 | -8.37 | **<0.001** | 95 | 0.00 | -8.44 | **<0.001** | 97 |
|  | Mass (mg) | 136.00 | -8.20 | **<0.001** | 108 | 560.00 | -4.20 | **<0.001** | 95 | 7.00 | -8.38 | **<0.001** | 97 |
|  | Age (dah) | 0.00 | -8.97 | **<0.001** | 108 | 0.00 | -8.35 | **<0.001** | 95 | 0.00 | -8.42 | **<0.001** | 97 |
|  | Standard metabolic rate (ml O_2_/h/mg) | 86.00 | -5.72 | **<0.001** | 64 | 450.00 | -0.83 | 0.405 | 64 | 92.00 | -5.64 | **<0.001** | 64 |
|  | Body condition | 0.00 | -8.95 | **<0.001** | 108 | 95.00 | -7.63 | **<0.001** | 95 | 0.00 | -8.41 | **<0.001** | 97 |
|  | Resting heart rate (bpm) | 0.00 | -6.91 | **<0.001** | 64 | 0.00 | -6.91 | **<0.001** | 64 | 0.00 | -6.89 | **<0.001** | 64 |
|  | Survival (%) | 5.50 | -0.83 | **0.405** | NA | 0.00 | -2.35 | **0.019** | NA | 0.00 | -2.39 | **0.017** | NA |
| **7** | Snout-vent length (mm) | 148.50 | -6.65 | **<0.001** | 85 | 0.00 | -7.36 | **<0.001** | 73 | 0.00 | -7.64 | **<0.001** | 80 |
|  | Mass (mg) | 146.00 | -6.79 | **<0.001** | 85 | 335.00 | -3.73 | **<0.001** | 73 | 22.50 | -7.51 | **<0.001** | 80 |
|  | Age (dah) | 0.00 | -7.92 | **<0.001** | 85 | 0.00 | -7.34 | **<0.001** | 73 | 0.00 | -7.62 | **<0.001** | 80 |
|  | Standard metabolic rate (ml O_2_/h/mg) | 115.50 | 5.34 | **<0.001** | 64 | 489.50 | -0.30 | 0.762 | 64 | 212.50 | -4.03 | **<0.001** | 64 |
|  | Body condition | 27.00 | -7.67 | **<0.001** | 85 | 296.00 | -4.06 | **<0.001** | 73 | 0.00 | -7.61 | **<0.001** | 80 |
|  | Resting heart rate (bpm) | 0.00 | -6.95 | **<0.001** | 64 | 0.00 | -6.95 | **<0.001** | 64 | 0.00 | -6.91 | **<0.001** | 64 |
|  | Survival (%) | 0.00 | -2.33 | **0.019** | NA | 4.00 | -1.18 | 0.237 | NA | 0.00 | -2.32 | **0.020** | NA |
|  | Relative fat body size in % body mass  (mg × ${mg body mass}^{-1}$) | 0.00 | -7.91 | **<0.001** | 85 | 0.00 | -7.33 | **<0.001** | 73 | 0.00 | -7.61 | **<0.001** | 80 |
